# Antipsychotic-induced prolactin elevation in premenopausal women with schizophrenia: associations with estrogen, disease severity and cognition

**DOI:** 10.1007/s00737-024-01491-9

**Published:** 2024-07-12

**Authors:** Bodyl A. Brand, Janna N. de Boer, Elske J. M. Willemse, Cynthia S. Weickert, Iris E. Sommer, Thomas W. Weickert

**Affiliations:** 1 Present Address: Department of Psychiatry, University of Oxford, Warneford Hospital, Oxford, OX3 7JX UK; 2 Department of Psychiatry, UMC Utrecht Brain Center, University Medical Center Utrecht (UMCU), Utrecht University, Utrecht, The Netherlands; 3 Present Address: Department of Biomedical Sciences and Systems, Cognitive Neurosciences, University of Groningen, University Medical Center Groningen (UMCG), Groningen, The Netherlands; 4 Neuroscience Research Australia, Sydney, NSW Australia; 5 Discipline of Psychiatry and Mental Health, University of New South Wales, Sydney, Australia; 6 Department of Neuroscience and Physiology, SUNY Upstate Medical University, Syracuse, NY USA; 7 Center for Young Children, Karakter Child and Adolescent Psychiatry, Nijmegen, The Netherlands

**Keywords:** Schizophrenia, Estrogen, Prolactin, Premenopausal women, Antipsychotics

## Abstract

**Purpose:**

Antipsychotic-induced prolactin elevation may impede protective effects of estrogens in women with schizophrenia-spectrum disorders (SSD). Our study sought to confirm whether the use of prolactin-raising antipsychotics is associated with lower estrogen levels, and to investigate how estrogen and prolactin levels relate to symptom severity and cognition in premenopausal women with SSD.

**Methods:**

This cross-sectional study included 79 premenopausal women, divided in three groups of women with SSD treated with prolactin-sparing antipsychotics (*n =* 21) or prolactin-raising antipsychotics (*n =* 27), and age-matched women without SSD (*n =* 31). Circulating 17β-estradiol was compared among groups. In patients, we assessed the relationship between prolactin and 17β-estradiol, and the relationships of these hormones to symptom severity and cognition, using correlation analyses and backward regression models.

**Results:**

In women receiving prolactin-raising antipsychotics, 17β-estradiol levels were lower as compared to both other groups (*H(*2) = 8.34; *p* = 0.015), and prolactin was inversely correlated with 17β-estradiol (*r*=-0.42, *p* = 0.030). In the prolactin-raising group, 17β-estradiol correlated positively with verbal fluency (*r* = 0.52, *p* = 0.009), and 17β-estradiol and prolactin together explained 29% of the variation in processing speed (*β*_17β−estradiol_ = 0.24, *β*_prolactin _= -0.45, *F*(2,25) = 5.98, *p* = 0.008). In the prolactin-sparing group, 17β-estradiol correlated negatively with depression/anxiety (*r *= -0.57, *p* = 0.014), and together with prolactin explained 26% of the variation in total symptoms (*β*_17β−estradiol_ = -0.41, *β*_prolactin_ = 0.32, *F*(2,18) = 4.44, *p* = 0.027).

**Conclusions:**

In women with SSD, antipsychotic-induced prolactin elevation was related to lower estrogen levels. Further, estrogens negatively correlated with symptom severity and positively with cognition, whereas prolactin levels correlated negatively with cognition. Our findings stress the clinical importance of maintaining healthy levels of prolactin and estrogens in women with SSD.

**Supplementary Information:**

The online version contains supplementary material available at 10.1007/s00737-024-01491-9.

## Introduction

High prolactin levels are often reported in chronically ill patients with schizophrenia-spectrum disorders (SSD) as a side-effect of antipsychotic medication (Peuskens et al. 2014). Many antipsychotics (e.g., most first-generation antipsychotics and several second generation antipsychotics) elevate prolactin levels in a dose-dependent manner by limiting the inhibitory effect of dopamine on prolactin secretion. Other second generation antipsychotics (e.g., clozapine and quetiapine) and third generation antipsychotics (aripiprazole, brexpiprazole, and cariprazine) induce only mild prolactin increases or do not raise prolactin at all (Peuskens et al. 2014). Women naturally have higher prolactin levels than men, and women with SSD receiving antipsychotic medication tend to have higher antipsychotic plasma concentrations as they often receive a dosage that may exceed their actual needs, due to sex differences in pharmacokinetic and pharmacodynamic processes (Jönsson et al. 2019; Ferrara and Srihari 2020; Brand et al. 2022). Women on antipsychotics are therefore more vulnerable to hyperprolactinemia than men, with a prevalence of 42–93% in women versus 18–72% in men (Bushe et al. 2008). Increased prolactin secretion can suppress gonadal sex hormones (i.e., predominantly estrogens in women and testosterone in men) (Riecher-Rössler 2017). The prevalence of this hypogonadal state is also more common in women with SSD (Smith et al. 2002b), which makes them susceptible for many negative health effects, such as sexual and menstrual dysfunction, osteoporosis, early menopause, and increased risk of breast cancer (Smith et al. 2002a; Markham 2012; González-Rodríguez et al. 2020; Taipale et al. 2021; Brand et al. 2022).

The consequences of prolactin-raising antipsychotics may not be limited to somatic health effects, but may also be of clinical relevance for women with SSD. On a neurobiological level, estrogens decrease neuro-inflammation, act as antioxidants, promote synaptic plasticity, and influence major neurotransmitter systems relevant for SSD, such as dopamine neurotransmission (Barth et al. 2015). Premenopausal women with SSD have the advantage of these neuroprotective actions of estrogens. Their naturally higher levels of estrogens are thought to explain why they experience fewer cognitive impairments and lower symptom severity at disease onset than men with SSD (Reilly et al. 2020; Brand et al. 2021). Estrogens enhance dopamine sensitivity of dopamine D2/D3 receptors in the ventral tegmental area (VTA) (Vandegrift et al. 2017). The VTA is integral to the mesolimbic and mesocortical pathway, linked respectively to positive and to negative and cognitive symptoms (Lewis and Lieberman 2000; Weinstein et al. 2017). There is evidence that women with SSD have lower estrogen levels than those without SSD (Riecher-Rossler et al. 1994; da Silva and Ravindran 2015; Meltzer-Brody et al. 2018; Brand et al. 2021; Hazelgrove et al. 2021). Moreover, women with SSD are increasingly vulnerable to psychosis onset and relapse, and experience more severe symptoms at times when estrogen levels are low, for example during the perimenstrual phase of their cycle (Reilly et al. 2020), after giving birth (Meltzer-Brody et al. 2018; Hazelgrove et al. 2021), and after menopause (Sommer et al. 2022). In women with SSD, low levels of estrogen are additionally linked to decreased cognitive functioning (Hoff et al. 2001; Ko et al. 2006; Yuan et al. 2016), and menstrual cycle irregularity has been found to predict poorer cognitive performance (Gurvich et al. 2018). Hypothetically, antipsychotic-induced suppression of endogenous estrogen production may have similar negative consequences on the course of disease in women with SSD.

Antipsychotic-induced prolactin elevation may cause a worsening of the clinical picture via its negative effect on a woman’s endogenous level of estrogens, as symptoms may be more resistant to improvement due to treatment with prolactin-raising antipsychotics. While some studies reported that high prolactin levels co-occurred with lower estrogen levels in women with SSD (Canuso et al. 2002; Bergemann et al. 2005, 2007), and another study showed inverse relationships between estrogens and prolactin levels (Smith et al. 2002b), it remains challenging to disentangle antipsychotic-induced hormone aberrations from those inherent to the clinical course of SSD in premenopausal women (Huber et al. 2001; Riecher-Rössler 2017). Studies that compared estrogen levels between women receiving prolactin-raising antipsychotics, women receiving prolactin-sparing antipsychotics, and women without SSD, have reported lower estrogen levels in women with SSD regardless of the group they belonged to (Canuso et al. 2002; Bergemann et al. 2005). Yet, sample sizes of subgroups were small, and the classification of prolactin-sparing and -raising antipsychotics does not corroborate with current literature (Peuskens et al. 2014; Huhn et al. 2019). Moreover, the extent to which antipsychotic-induced prolactin elevation and estrogen deficiency are related to symptom severity and cognition remains unestablished.

The current study sought to confirm whether the use of prolactin-raising antipsychotics is associated with increased circulating prolactin and reduced estrogen (17β-estradiol), and whether these hormone alterations are related to higher symptom severity and more impaired cognitive functioning in premenopausal women with SSD. To disentangle antipsychotic-induced hormone alterations from those that may be inherent to SSD itself, a sample of women receiving prolactin-sparing antipsychotics and a sample of women receiving prolactin-raising antipsychotics are compared. In addition, we include a sample of women without SSD for comparison.

## Materials and methods

### Study design

This cross-sectional study of premenopausal women with and without SSD combined baseline (pre-treatment) data of a European clinical trial in men and women with SSD (Brand et al. 2020), and an Australian clinical trial in patients with SSD, with a comparator group of individuals without SSD (control group) (Weickert et al. 2015). From these study-samples, we selected the premenopausal participants (Peuskens et al. 2014). Premenopausal status was established by querying the patient about the regularity of their menstrual cycle, age, and confirmed through the assessment of FSH levels (< 25 IU/L). Participants were ⩾18 years of age. Presence or absence of psychopathology was established using The Mini International Neuropsychiatric Interview 5.0 (Overbeek et al. 1999) or the Structured Clinical Interview (First 2007). All patients used at least one type of antipsychotic medication. Exclusion criteria for all participants were the use other prolactin-raising medications, history of pre-existing cardiovascular disease, uncontrolled diabetes or hypertension, liver function or enzyme disorders, pregnancy, or breastfeeding. Procedures are described in more detail in the eMethods in the supplementary.

### Hormonal assays

Fasting peripheral blood was obtained through venepuncture between 9:00 and 11:30 am. Levels of prolactin and 17β-estradiol were assessed using electrochemiluminescence enzyme immunoassay (ECLIA) using the Siemens Immulite 2000 (Australian women) or the Atellica IM Analyzer (European women). For the Australian sample, the 17β-estradiol interassay coefficient of variability (CV) was 13.5% and the intra- and interassay CVs for prolactin were 3.4% and 6.8%, respectively. For the European sample, interassay CVs were 7.4% for 17β-estradiol and 3.1% for prolactin. A prolactin concentration > 530 mIU/L (> 25 ng/mL) was set as the cutoff for hyperprolactinemia (Peuskens et al. 2014). For three healthy controls and six patients, samples had 17β-estradiol levels at or below the sensitivity limit of 70 pmol/L. These very low 17β-estradiol values were set to 35 pmol/L.

### Antipsychotic medication

Based on previously published antipsychotic clinical guides (Peuskens et al. 2014; Huhn et al. 2019), participants were assigned to the ‘prolactin-sparing group’ (aripiprazole, brexpiprazole, cariprazine, clozapine, flupenthixol, and quetiapine) or ‘prolactin-raising group’ (haloperidol, olanzapine, paliperidone, risperidone, ziprasidone, zuclopenthixol, or polytherapy with at least one prolactin-raising antipsychotic). One woman used haloperidol and aripiprazole and was assigned to the prolactin-sparing group as aripiprazole addition reduces the prolactin-raising effect of other antipsychotics (Labad et al. 2020).

### Clinical assessments

The Positive and Negative Syndrome Scale (PANSS) was assessed by a trained research assistant or psychologist to measure current symptom severity (Kay et al. 1987). The total PANSS-score reflects the severity of the disease (see eMethods). Five factors were calculated to indicate the severity of positive, negative, cognitive, excitement/hostility, and depression/anxiety symptoms (Citrome et al. 2011). Cognition was assessed using either the Wechsler Adult Intelligence Scale-3rd edition (WAIS-III: Wechsler, 1997) for the Australian sample, and the Brief Assessment of Cognition in Schizophrenia (BACS: Keefe et al. 2004) for the European sample. Cognition scores were combined into three cognitive domains: processing speed, verbal fluency and working memory (see eTable 3). Consequently, cognitive test scores were converted into age- and sex-corrected Z-scores to enable comparisons across the two studies. Illness duration and age at onset were calculated based on the age at first diagnosis. The highest educational level achieved was converted into the number of years of education (see eTable 4).

### Statistical analyses

Statistical analyses were performed in R version 4.2.0. First, age, prolactin, 17β-estradiol, and cognition scores were compared among the three groups using one-way ANOVA or the non-parametric equivalent Kruskall-Wallis *H* Test. Pairwise post-hoc comparisons were performed using Tukey’s HSD or Dunn’s Test, with Benjamin-Hochberg False Discovery Rate (FDR) corrections. Second, PANSS-scores were compared between the prolactin-raising and prolactin-sparing group using t-tests or a non-parametric equivalent. Fishers’s exact Test was used for dichotomous variables. Third, we explored the relationship between prolactin and 17β-estradiol in the prolactin-raising and prolactin-sparing groups separately (Pearson’s correlation coefficient, *r*). When necessary, prolactin and 17β-estradiol were transformed. Similarly, prolactin and 17β-estradiol and relationships between these hormones were linked to cognition scores and symptom scores. *P-*values were FDR-corrected. Finally, to determine the extent to which each hormone explains variability in symptoms and cognition, backward regression analyses were performed for outcome measures showing moderate correlations (*r* ≥ 0.40 or *r≤*-0.40) with both hormones. We checked for potential confounding associations between hormone levels and demographic variables (age, daily olanzapine-equivalent dose, age at onset). Sensitivity analyses were performed by excluding all hormonal contraception users from analyses.

## Results

### Sample description

Demographics and serum hormone levels of patients using prolactin-sparing antipsychotics (*n* = 21), patients using prolactin-raising antipsychotics (*n* = 27) and unmedicated controls (*n* = 31) are provided in Table 1. Clinical characteristics of the two patient groups are provided in Table 2. Australian patients (*n* = 35) and European patients (*n* = 13) did not differ significantly in terms of demographic or clinical variables (eTable 5). Most women in the prolactin-sparing group received monotherapy with aripiprazole (38%, *n =* 8) or clozapine (38%, *n =* 8, eTable 6). Women in the prolactin-raising group mostly received olanzapine monotherapy (22%, *n =* 6), or polypharmacy with at least one prolactin-raising antipsychotic and without aripiprazole (30%, *n =* 8). Use of hormonal contraception was less common in the prolactin-raising group, as compared to the other two groups (Fisher’s Exact Test, *p =* 0.008, Table 1). There were no differences in clinical characteristics between patient groups (all *p*’s > 0.05).

**Table 1:** Demographics and serum hormone levels of patients and controls

	Patients		Controls
	Prolactin-sparing group	Prolactin-raising group	
	*n =* 21	*n =* 27	*n* = 31
Age in years, mean (SD)	36.0 (8.90)	36.4 (8.40)	32.8 (6.40)
Prolactin in mIU/L, median [min, max]	180 [25.0, 540]^a^	876 [72.0, 2350]^a, b^	172 [60.0, 427]^b^
17β-oestradiol in pmol/L, median [min, max]	283 [35.0, 2200]^a^	168 [35.0, 1260]^a, b^	284 [35.0, 2010]^b^
Hyperprolactinemia, *n* (%)	1 (5%)	16 (59%)	0 (0%)
Information on contraceptive use, *n*	21	27	27
Contraceptive use, *n* (%)	8 (38%)	2 (7%)^c^	11 (41%)
Information on menses available^1^, *n*	12	19	13
Irregular menses, *n* (%)	1 (8%)	9 (47%)^d^	1 (8%)

SSD, Schizophrenia-Spectrum Disorder; SD, Standard Deviation

^1^of patients without contraceptive use

^a^significant difference between prolactin-sparing and prolactin-raising group (adjusted *p* < 0.05)

^b^significant difference between prolactin-raising and control group (adjusted *p* < 0.05)

^c^significantly lower proportion as compared to the prolactin-sparing group and group of women without SSD (*p* < 0.05)

^d^significantly higher proportion as compared to the prolactin-sparing group and group of women without SSD (*p* < 0.05)

### Influence of antipsychotic therapy on hormone levels

**Fig. 1:**
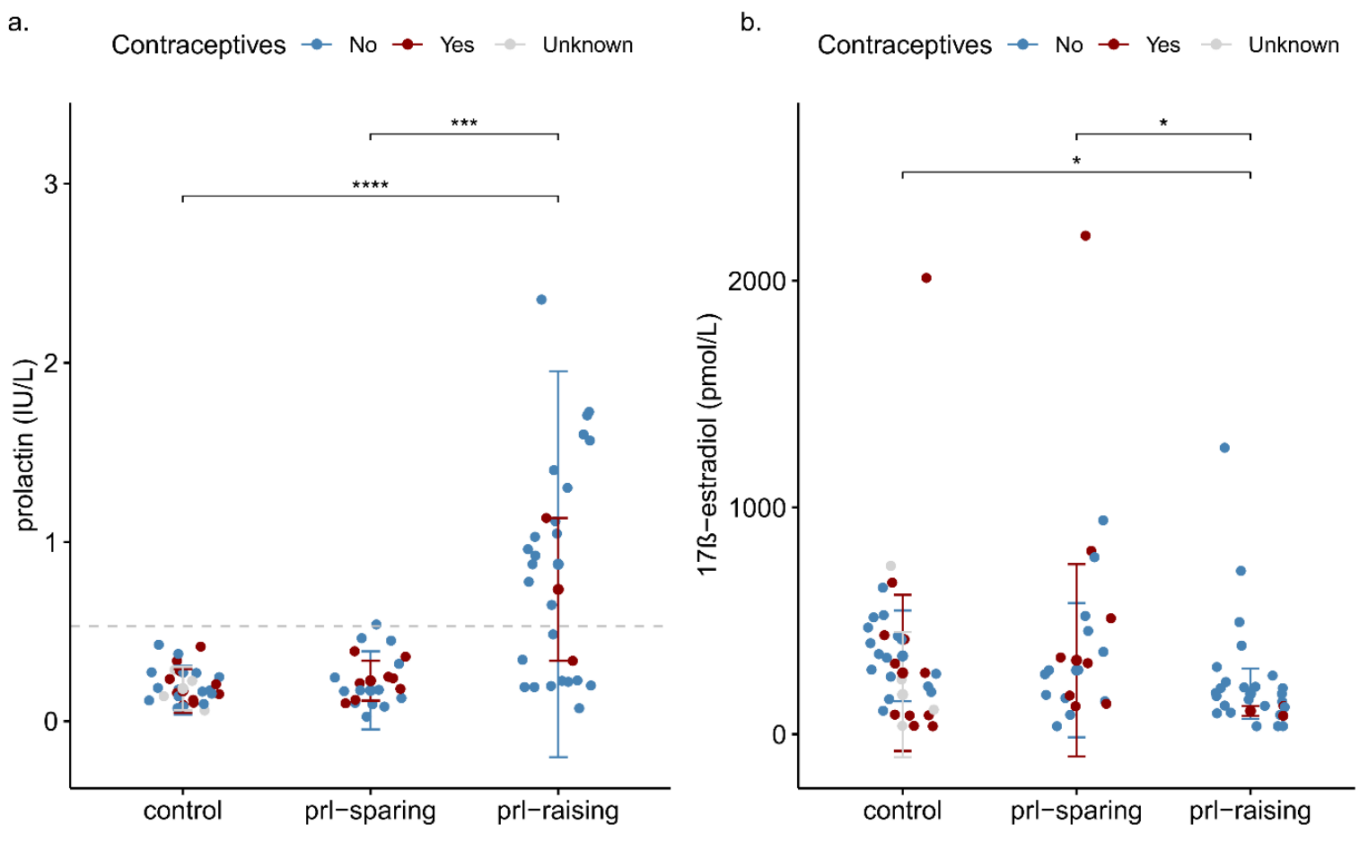
Group comparisons of prolactin levels (1**a**) and 17β-estradiol levels (1**b**). Prolactin levels and 17β-estradiol with median and IQR of control group (*n* = 31), the prolactin-sparing group (*n =* 21) and the prolactin-raising group (*n* = 27). The dashed horizontal line in 1a represents the hyperprolactinemia cut-off (> 0.530 IU/L). * adjusted *p <* 0.05, ***adjusted *p <* 0.0005, ****adjusted *p* < 0.00005

Groups differed in both prolactin (*H*(2) = 24.98; *p* < 0.001), and 17β-estradiol levels (*H*(2) = 6.74; *p* = 0.034; Table 1; Fig. 1). Post-hoc analyses revealed higher prolactin levels and lower 17β-estradiol levels in the prolactin-raising group as compared to controls (*p* < 0.001 for prolactin; *p* = 0.049 for 17β-estradiol), and the prolactin-sparing group (*p* < 0.001 for prolactin; *p =* 0.049 for 17β-estradiol). Hyperprolactinemia (> 530 mIU/L) was detected in one woman (5%) in the prolactin-sparing group and in 16 women (59%) in the prolactin-raising group (Fig. 1). Among women without hormonal contraception, the prevalence of irregular menses was significantly higher in the prolactin-raising group (Fishers Exact Test *p =* 0.014) (Table 1).

### Associations of hormones with symptom severity and cognitive functioning

**Fig. 2:**
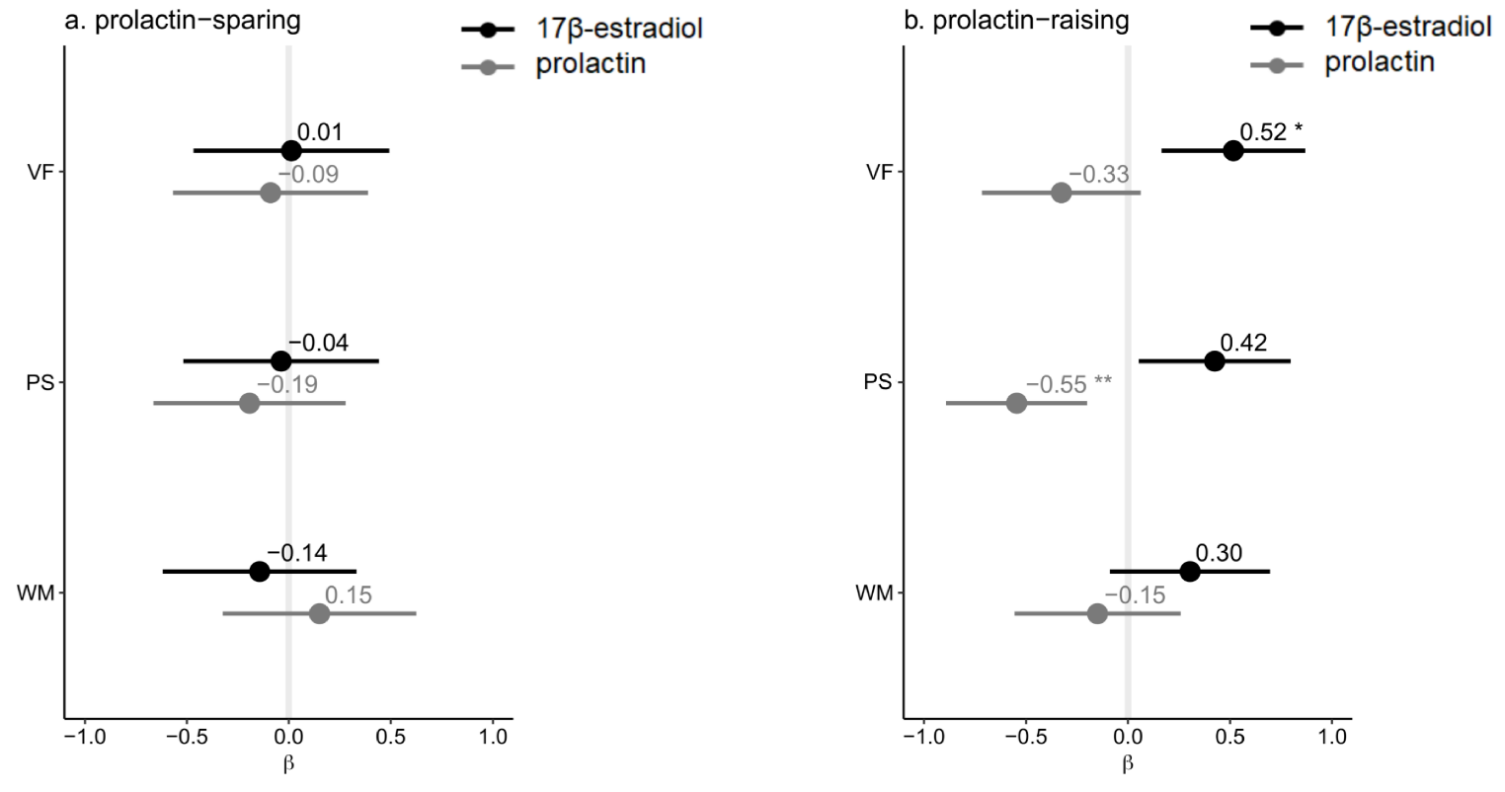
Forest plots of correlations between hormone levels and cognition scores in patients receiving prolactin-sparing antipsychotics (**a**) and in patients receiving prolactin-raising antipsychotics (**b**) Pearson correlation coefficients (*r*) with 95% CI’s between log-transformed levels of 17β-estradiol (black) and prolactin (grey) on the one hand and cognition scores on the other hand. VF, verbal fluency; PS, processing speed; WM, working memory. *adjusted *p <* 0.05, **adjusted *p <* 0.01

**Fig. 3:**
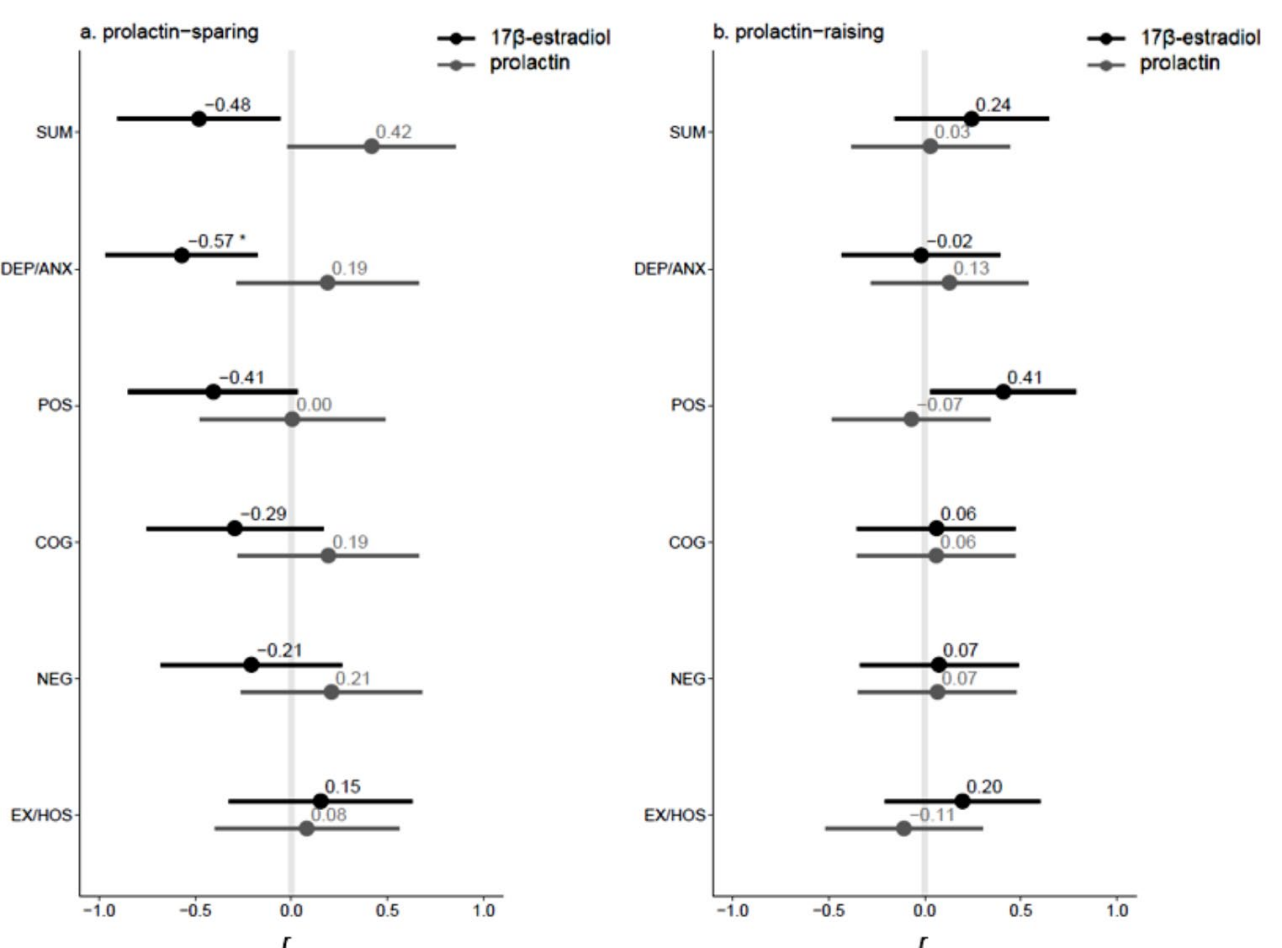
Forest plots of correlations between hormone levels and symptom severity scores in patients receiving prolactin-sparing antipsychotics (**a**) and in patients receiving prolactin-raising antipsychotics (**b**) Pearson correlation coefficients (*r*) with 95% CI’s between log-transformed levels of 17β-estradiol (black) and prolactin (grey) and PANSS symptom severity scores on the other hand. SUM, total symptom severity; DEP/ANX, depression/anxiety factor; POS, positive factor; COG, cognitive factor; NEG, negative factor; EX/HOS, excitement/hostility factor. *adjusted *p* < 0.05

We found a significant negative association between prolactin and 17β-estradiol levels in the prolactin-raising group (*r*(25) = -0.42, *p =* 0.030), but not in the prolactin-sparing group (*r*(19) = -0.23, *p =* 0.32) nor in women without SSD (*r*(29) = 0.058, *p* = 0.76; eTable 7). Prolactin and/or 17β-estradiol were not correlated with age, duration of illness, age at onset, or daily olanzapine equivalents (all *p*’s > 0.10; eTable 8). In the prolactin-raising group, we found significant correlations of 17β-estradiol with verbal fluency and processing speed (Fig. 2a). In this group, lower 17β-estradiol was significantly associated with lower verbal fluency (*r*(25) = 0.52, *p =* 0.009) and lower processing speed scores (*r*(25) = 0.42, *p =* 0.054). Additionally, we found a significant inverse correlation between prolactin and processing speed (*r*(25) = -0.55, *p =* 0.006). In the prolactin-sparing group, higher 17β-estradiol levels were associated with lower scores on the PANSS depression/anxiety scores (*r*(19) = -0.57, *p =* 0.014; Fig. 3b). Similar inverse correlations in the prolactin-sparing group between 17β-estradiol and PANSS positive scores (r(19) = -0.41, *p =* 0.13) and PANSS total scores (*r*(19)=-0.48, *p =* 0.055), and a positive correlation between prolactin and PANSS total scores (*r*(19) = 0.42, *p =* 0.11), did not reach significance.

### Combined effects of prolactin and 17β-estradiol on symptom severity and cognitive functioning

To determine the extent to which prolactin and 17β-estradiol explain variability in symptoms and cognition, backward regression analyses were performed for processing speed in the prolactin-raising group, and for PANSS total scores in the prolactin-sparing group. In the prolactin-raising group, processing speed was more strongly predicted by prolactin (*β *= -0.45, *p =* 0.022) than by 17β-estradiol (*β* = 0.24, *p =* 0.20, eTable 9), and together, they explained a 29% of the variance in processing speed scores (adjusted *R*² = 0.29, *F*(2,24) = 6.31, *p =* 0.006). In the prolactin-sparing group, PANSS total scores were more strongly predicted by 17β-estradiol (*β *= -0.41, *p =* 0.055) than by prolactin (*β* = 0.32, *p =* 0.12), together explaining 26% of the variance (adjusted *R*² = 0.26, *F*(2,18) = 4.44, *p =* 0.027).

### Sensitivity analyses

As a sensitivity analysis, we repeated all analyses after excluding women using hormonal contraception. Group differences between the control and prolactin-raising group remained significant for prolactin (*p* < 0.001) and 17β-estradiol (*p* = 0.017), whereas group differences between the prolactin-sparing and prolactin-raising group remained significant for prolactin (*p* < 0.001), but not for 17β-estradiol (*p* = 0.14) (eTable 11, eFigure 4). No significant group differences were found between the prolactin-raising and prolactin-sparing group regarding any clinical characteristics (eTable 12). All correlation and regression analyses yielded similar results to analyses based on the whole sample (eTable 7, eTable 9, eFigure 5–6).

**Table 2:** Clinical characteristics of patients

	All patients	Prolactin-sparing group	Prolactin-raising group
	*n =* 48	*n =* 21	*n =* 27
Daily olanzapine equivalent dose in mg/day, mean (SD)	19.2 (15.3)	19.0 (18.6)	19.3 (12.3)
Illness duration in years, median [min, max]	11.0 [3.00, 40.0]	11.0 [3.0, 36]	11.0 [3.00, 40.0]
Age at onset in years, mean (SD)	22.6 (6.59)	22.9 (6.67)	22.5 (6.65)
Education in years, mean (SD)	11.9 (2.32)	12.2 (2.45)	11.6 (2.22)
PANSS total score, mean (SD)	56.2 (12.8)	56.6 (13.6)	55.8 (12.4)
Positive factor, mean (SD)	12.0 (5.06)	11.9 (5.08)	12.1 (5.14)
Negative factor, mean (SD)	9.46 (3.95)	9.52 (4.24)	9.41 (3.79)
Cognitive factor, mean (SD)	15.5 (4.76)	16.2 (5.36)	15.0 (4.27)
Excitement/Hostility factor, mean (SD)	5.38 (1.93)	5.00 (1.26)	5.67 (2.30)
Depression/Anxiety factor, mean (SD)	13.3 (3.64)	12.7 (3.66)	13.7 (3.64)
Processing speed z-score, mean (SD)	-1.53 (0.837)	-1.52 (0.960)	-1.54 (0.747)
Verbal fluency z-score, mean (SD)	-0.896 (1.17)	-1.05 (1.16)	-0.779 (1.19)
Working memory z-score, mean (SD)	-1.10 (0.987)	-0.967 (0.757)	-1.21 (1.14)

SSD, Schizophrenia-Spectrum Disorder; PANSS, Positive and Negative Syndrome Scale; SD, Standard Deviation

### Discussion and conclusions

Our findings showed that antipsychotic-induced prolactin elevation was related to lower estrogen levels, and that estrogen levels correlated negatively with symptom severity and positively with cognition in women with SSD. In women receiving prolactin-sparing antipsychotics, estrogen correlated negatively with depression/anxiety symptoms. The stronger impact of higher estrogen levels rather than lower prolactin levels on total symptom severity highlight the importance of maintaining healthy estrogen levels, and is consistent with studies reporting that higher levels of estrogens are associated with lower disease severity (Reilly et al. 2020; Brand et al. 2021; Sommer et al. 2023), and with RCTs reporting beneficial effects of estrogen augmentation on symptom severity in women with SSD (for a meta-analysis, see Zijia Li et al. 2023). In the prolactin-raising group, prolactin levels were higher, estrogen levels were lower, and prolactin was inversely correlated with estrogen, suggesting that lower estrogen levels may not be inherent to the disorder itself but related to prolactin-raising antipsychotic treatment. Moreover, estrogen correlated positively with verbal fluency, and high levels of prolactin negatively impacted processing speed. These results support the notion that prolactin-raising antipsychotics can inhibit estrogen production, and both elevated prolactin and low estrogen may have adverse effects on cognitive function (Riecher-Rössler 2017; Kulkarni et al. 2019). While women may still benefit from the protective effects of endogenous estrogens when using prolactin-sparing antipsychotics, they may be deprived from of the full protective effects of estrogens on psychotic symptoms and cognition when using prolactin-raising antipsychotics. After controlling for contraceptive use, trends in group differences and associations were mainly consistent, yet some comparisons no longer reached statistical significance. This might be attributed to the relatively limited sample sizes, although the possible influence of contraceptive use cannot be excluded.

To our knowledge, this is the first study that thoroughly investigated the hormonal and clinical effects of antipsychotic-induced prolactin elevation in premenopausal women with SSD. Our findings indicate that the potential negative consequences of prolactin-raising antipsychotics should be considered in the treatment of premenopausal women with SSD and stress the clinical relevance of maintaining healthy levels of prolactin and estrogen in women with SSD. Screening of prolactin levels during antipsychotic treatment has already been recommended since 2008, and yet, it has not been translated into clear clinical guidelines to date (Grigg et al. 2017). Our findings emphasize the need for such guidelines, to assist clinician and patient decision-making to avoid prolactin elevation whenever possible. Our findings of a strong association of estrogens with symptoms suggest maintaining a menstrual symptom diary can provide insights regarding the interplay between estrogen and symptoms, which may inform treatment adjustments on a patient-level.

In women using prolactin-sparing antipsychotics, our findings of positive associations of estrogens and negative associations of prolactin with cognitive functioning suggest that in this group of patients, both lower estrogen levels and higher prolactin levels may impact cognitive functioning. In men, a similar complex relationship of prolactin and testosterone with cognition may exist, with some studies relating impaired cognition to elevated prolactin (Bratek et al. 2015; Montalvo et al. 2018; Hidalgo-Figueroa et al. 2022), while others report more consistent positive associations of testosterone with cognition (Moore et al. 2013). Findings on the associations of estrogens with cognition studies in women with SSD are inconsistent, as some align with our findings (Hoff et al. 2001; Ko et al. 2006; Yuan et al. 2016) while others do not (Halari et al. 2004; Rubin et al. 2015; Yuan et al. 2016; Montalvo et al. 2018). Importantly, our findings corroborate with Ko et al. (2006), who included a study sample most similar to ours (i.e., premenopausal SSD patients with low levels of estrogens) (Ko et al. 2006). Our findings also support the notion that in healthy women, menopause-associated estrogen decline is associated with cognitive impairment (Hogervorst et al. 2022), and that this can be reversed by estrogen replacement therapy (Sherwin and Tulandi 1996; Verghese et al. 2000). Studies on augmentation with estrogen or estrogen-like compounds in women with SSD have shown mixed results, as some reported positive effects on cognitive function (Huerta-Ramos et al. 2014; Weickert et al. 2015; Brand et al. 2023), while others did not (Kulkarni et al. 2015; Weiser et al. 2017, 2019; Huerta-Ramos et al. 2020). Associations of prolactin with cognition may be mediated by distinct actions of prolactin-raising antipsychotics on brain regions involved in cognition. However, our results align both with studies in female SSD patients that were medicated (Ko et al. 2006), unmedicated (Yuan et al. 2016), and partly unmedicated (Montalvo et al. 2018; Hidalgo-Figueroa et al. 2022), suggesting that cognition may be affected by elevated prolactin levels independently from the effects of antipsychotics on cognition through other routes. In sum, future research should further establish the interplay between age, prolactin, estrogens, antipsychotic treatment status and type, and cognition in women and in men with SSD.

There are several ways in which hyperprolactinemia can either be prevented or mitigated. When prescribing antipsychotics, prolactin-sparing antipsychotics should be preferred over prolactin-raising ones. When prescribing prolactin-raising antipsychotics is unavoidable, careful monitoring of hormone-related side-effects and prolactin levels is recommended. It should however be noted that for some antipsychotics, prolactin elevation can be transient, with prolactin levels normalizing within one year (Peuskens et al. 2014). In the case of hyperprolactinemia, inquiring thoroughly about its associated side-effects is essential, and strategies to reduce prolactin levels should be considered (i.e. dose reduction, switching to an alternative antipsychotic or adding aripiprazole) (Zhang et al. 2021; Taylor et al. 2021; Lu et al. 2022).

### Limitations

A major limitation of this cross-sectional study is that it cannot establish a causal link between hormone levels and clinical measures. Another limitation concerns the difficulty in attributing associations of hormone levels with cognition solely to the prolactin-raising nature of antipsychotics, as many of the effects of prolactin-raising antipsychotics are also likely mediated by a distinct and more direct action on brain regions involved in cognition, such as the cortex and striatum. Given the many different factors influence cognition in women with SSD, a multivariate analysis approach that includes multiple hormone levels and different antipsychotic types with a larger sample size could be designed to confirm and extend our findings. In addition, our approach of dividing patients in into two groups, i.e., the prolactin-raising or prolactin-sparing group, may be overly simplistic as it does not adequately capture the complexity of determining whether a medication increases prolactin levels. Prolactin elevation can be influenced by many factors such as dosage, treatment duration, and baseline prolactin levels (Peuskens et al. 2014). Furthermore, the determination of prolactin levels from our assays may have been influenced by venipuncture stress and the pulsatile nature of prolactin secretion (Melmed et al. 2011; Whyte et al. 2015). Although we minimized the impact of the circadian rhythm of prolactin secretion by timing the blood tests within a period when prolactin levels are relatively stable (Roelfsema et al. 2012), we were unable to reduce or eliminate the potential impact of venipuncture stress (Whyte et al. 2015). In addition, timing of the hormonal assessments relative to the menstrual cycle brings several challenges, as estrogen levels are known to fluctuate across the cycle (Hampson 2020). Although all women should preferably be assessed at the same time in the cycle, within every woman, the timing of individual events and the hormone levels achieved at each stage also varies significantly from cycle to cycle (Hampson 2020). Also, we could not account for the potential influence of progesterone, which may be difficult to disentangle from the effects of estrogen. While the production of progesterone is negligible during the first peak of estrogen in the follicular phase, it peaks together with estrogen during the luteal phase (Sun et al. 2016). Thus, future studies on the action of progesterone and its interplay with estrogens are needed to establish their complex interplay, for example by comparing associations of both sex hormones with clinical outcomes in the follicular and luteal phase. Furthermore, the extent to which hormonal contraception affects endogenous hormone levels is unclear, also because these synthetic hormones are not detectable with standard immunoassays (Hampson 2020). Although similar results were found after excluding women using hormonal contraception, their potential influence on the association between hormones and outcome measures remains unclear and should be addressed by future studies in larger samples. Finally, there are some disadvantages associated with the use of immunoassays, including cross-reactivity with similar analytes, and standardization and sensitivity issues. These concerns are suggested to become specifically problematic when expected levels of estrogens fall below 70 pmol/L (Taylor et al. 2015),  whereas premenopausal women typically have estrogen levels above this threshold (Verdonk et al. 2019), and in our sample, only a small proportion of women had estrogen levels below this value.

## Conclusions

This study showed that antipsychotic-induced prolactin elevation was associated with lower levels of estrogen, higher symptom severity and lower cognitive functioning in premenopausal women with SSD. While these women should benefit from the protective action of endogenous estrogen, prolactin-raising antipsychotics may impede these benefits. Our findings emphasize the clinical importance of maintaining healthy prolactin and estrogen levels in premenopausal women with SSD.

## Electronic supplementary material

Below is the link to the electronic supplementary material.

Supplementary Material 1
