# Supplementary material for: Antipsychotic-induced prolactin elevation in premenopausal women with schizophrenia: associations with estrogen, disease severity and cognition
Source: Arch Womens Ment Health. 2024 Jul 12;27(6):931–41. doi: 10.1007/s00737-024-01491-9 (PMC11579114; doi:10.1007/s00737-024-01491-9)
Supplement: Supplementary file 1 — Supplementary Material 1 [file 737_2024_1491_MOESM1_ESM.docx]

**Comments for the Author:**

*Reviewer #5: The comments and related revisions are all very satisfactory.
The article is very much improved.*

*Reviewer #6: Dear authors, I think your study is very fruitful and have high quality for printing.*

1. *You'd better write all the antipsychotic(from prolacting racing group) names and (if there) long acting ones. Thank you for the study.*

We thank the reviewer for this suggestion. We have specified the prolactin-raising antipsychotics in line 120-121 of the manuscript and specified the use of oral and LAI antipsychotics in supplementary table 6.

*Reviewer #7: None

Reviewer #8: This paper presents the results of an interesting preliminary study of the relationship between serum prolactin and estrogen (17-beta estradiol) in women with schizophrenia spectrum disorders receiving various classes of antipsychotics, and their possible impact on symptomatology and cognition.

The rationale for the study has been stated clearly and the authors have laid a strong conceptual framework for undertaking this research. The methodology has been described in sufficient detail and is free of any major flaws. Overall, this study represents a potentially valuable contribution to the existing literature on antipsychotic-induced elevations in prolactin, and its focus on women (who are more vulnerable to adverse health outcomes related to hyperprolactinemia) is commendable.

The following are aspects of the paper that require a certain degree of correction or clarification:*

*1. There is some evidence that antipsychotic-induced elevations in prolactin may normalize over time (especially after the first 12 months of treatment), possibly due to receptor desensitization or downregulation. This should be taken into account when discussing the long-term effects of elevated prolactin on physical and mental health.*

We thank the reviewer for bringing this to our attention. We agree that it is important to acknowledge that antipsychotic-induced prolactin elevation can be transient for certain antipsychotics. While information regarding the duration of antipsychotic treatment was not available for our sample, the patients included in our study were relatively stable (i.e., there were no recent changes in antipsychotic medications) and had a duration of illness of three years or more. Therefore, we believe the likelihood of including many patients with transiently elevated prolactin levels is low.

In response to the reviewer's comment, we have made the following adjustments to the discussion:

Page 12, line 292-293:

…. prolactin-raising antipsychotics is unavoidable, careful monitoring of hormone-related side-effects and prolactin levels is recommended. It should however be noted that for some antipsychotics, prolactin elevation can be transient, with prolactin levels normalizing within one year (Peuskens et al., 2014). In the case of hyperprolactinemia….

Page 12, line 305-308:

…. a multivariate analysis approach that includes multiple hormone levels and different antipsychotic types with a larger sample size could be designed to confirm and extend our findings. In addition, our approach of dividing patients in into two groups, i.e., the prolactin-raising or prolactin-sparing group, may be overly simplistic as it does not adequately capture the complexity of determining whether a medication increases prolactin levels. Prolactin elevation can be influenced by many factors such as dosage, treatment duration, and baseline prolactin levels (Peuskens et al. 2014).

*2. The statement at lines 71-72 that "Antipsychotic-induced prolactin elevation may cause a worsening of the clinical picture via its negative effect on a woman's endogenous level of estrogens" is not clearly supported by the evidence, and the authors themselves acknowledge this on lines 82-83. This could be rephrased for the sake of clarity.*

We thank the reviewer for this suggestion. We aimed to emphasize the uncertainty of this effect by making the following adjustments:

Hypothetically, antipsychotic-induced suppression of endogenous estrogen production may thus have similar negative consequences on the course of disease in women with SSD.

*3. In lines 80-81, the authors raise the concern of false-negative findings in earlier studies due to small sample sizes. Is the current study adequately powered to address the primary objective? Details of study power / sample size calculation should be provided in the Methods section of the main text.*

We thank the reviewer for bringing this to our attention. In order to keep the analyzes as reliable and robust as possible given our small sample size, we aimed to limit the complexity of the analysis. For this reason, we utilized regression models only when our correlation analysis indicated that the effects of estradiol and prolactin were of similar strength and significance.

In response to the reviewer’s comment, we conducted post-hoc power analyses on the significant correlation tests and the two multiple linear regression models. For the correlation tests, power was calculated based on the sample size, correlation coefficient, and significance level, demonstrating a power ranging between 0.80 and 0.86. Regarding the regression models, we utilized the F-statistic and effect size (0.89 for processing speed model, 0.69 for PANSS total model), indicating a power of 0.86 and 0.67, respectively. These analyses indicate that our study was adequately powered to detect the observed effects, with a slightly lower but still sufficient power for the PANSS total regression model.

*4. The inclusion of flupentixol in the "prolactin-sparing group" is somewhat debatable. Although this drug causes less hyperprolactinemia than other typical antipsychotics, its prolactin-elevating properties may be more marked in women (Retief et al., 2019). Moreover, this drug is often administered in long-acting injectable (LAI) format which may result in significant differences in pharmacokinetics from oral medication. It might be useful to try analyzing the data twice (i.e., placing it in either group) to see if the study results differ accordingly.*

We acknowledge the reviewer's concerns regarding the potential prolactin-raising effect of flupentixol. Our categorization was based on the findings of Huhn et al., (2019), which indicated that flupentixol presents a lower risk of prolactin elevation when compared to placebo. Although we predetermined the groups based on these findings, we checked the prolactin levels of the subjects taking flupentixol, which were not increased. The two patients who received flupentixol in our sample had prolactin levels within the reference range (240 mIU/L for the participant receiving flupentixol orally, 320 mIU/L for the participant receiving flupentixol LAI).

While there is some indication that the prolactin-raising effect of LAIs may be slightly less than that of oral antipsychotics, the evidence for this is inconsistent and findings may be influenced by differences in actual serum concentrations (i.e., that those who received LAI received lower doses and therefore had lower prolactin levels) (Zhornitsky & Stip, 2012).

*4b. Likewise, the prolactin-sparing or -raising effects of antipsychotic drugs are often dose-dependent (e.g., risperidone causes less prolactin elevation when daily doses are <4 mg). Was any attempt made to convert the doses of medication into dose equivalents ("chlorpromazine equivalents") and factor this into the analyses?*

We acknowledge that dosage may influence the degree of prolactin elevation in our study sample. To investigate the potential associations between the dose of antipsychotics (converted to olanzapine equivalents) on the one hand and the hormone levels on the other, we performed correlation analyses, the results of which are described on page 6, lines 176-177 and in eTable 8 in the supplement. These analyses show that this effect was not present. The lack of this association may be explained by the fact that certain antipsychotics can cause hyperprolactinemia even at low doses (Haddad & Wieck, 2004). This poses challenges in detecting a relationship within study samples with many different types of antipsychotics.

*5. Was any specific battery or set of standardized tests used to assess cognition? This should be mentioned briefly in lines 127-128.*

In response to the reviewer, we have now specified the cognitive tests used in line 128-133 of the manuscript. It now reads as follows:

Five factors were calculated to indicate the severity of positive, negative, cognitive, excitement/hostility, and depression/anxiety symptoms (Citrome et al. 2011). Cognition was assessed using either the Wechsler Adult Intelligence Scale-3rd edition (WAIS-III: Wechsler, 1997) for the Australian sample, and the Brief Assessment of Cognition in Schizophrenia (BACS: Keefe et al., 2004) for the European sample. Cognition scores were combined into three cognitive domains: processing speed, verbal fluency and working memory (see eTable 3). Consequently, cognitive test scores were converted into age- and sex-corrected Z-scores to enable comparisons across the two studies.

*6. Given the relatively small sample sizes (and the need for non-parametric tests in some cases, line 133), is the Pearson's r appropriate to examine correlations between prolactin and estrogen levels? Would an alternate correlation test (Spearman's or Kendall's) have been preferrable?*

We transformed our data in order to meet the assumptions for a Pearson correlation (i.e. normality and homoscedasticity). This approach was preferred over using a non-parametric test without transformations, as a Pearson correlation with transformed data provides a more powerful and precise measurement of the relationship between variables when assumptions of normality and homoscedasticity are met. In contrast, nonparametric tests with untransformed data are robust but less powerful.

*7. In Table 1, the proportion of women experiencing irregular menses in Group 2 is given as "9 (4%)". Should this be corrected to 9 (33%) as the subgroup includes only 27 individuals?*

We thank the reviewer for bringing this inaccuracy to our attention. The proportion of irregular menses should be 47% instead of 4%. We calculated this proportion based on the number of participants for whom information on menses was available in this group (*n*=19).

**References**

Haddad, P. M., & Wieck, A. (2004). Antipsychotic-induced hyperprolactinaemia: mechanisms, clinical features and management. *Drugs*, *64*, 2291–2314. doi: 10.2165/00003495-200464200-00003

Huhn, M., Nikolakopoulou, A., Schneider-Thoma, J., Krause, M., Samara, M., Peter, N., … Leucht, S. (2019). Comparative efficacy and tolerability of 32 oral antipsychotics for the acute treatment of adults with multi-episode schizophrenia: a systematic review and network meta-analysis. *Lancet*, *394*(10202), 939–951. doi: 10.1016/S0140-6736(19)31135-3

Zhornitsky, S., & Stip, E. (2012). Oral versus long-acting injectable antipsychotics in the treatment of schizophrenia and special populations at risk for treatment nonadherence: A systematic review. *Schizophrenia Research and Treatment*, *2012*, 407171. doi: 10.1155/2012/407171
